# Double Dislocation of Proximal and Distal Interphalangeal Joints With Volar Plate Avulsion Fracture in the Fifth Digit of an Elderly Female: Successful Conservative Management

**DOI:** 10.7759/cureus.114370

**Published:** 2026-08-11

**Authors:** Claire Chaljub, Natalie R Black, John J Faillace

**Affiliations:** 1 School of Medicine, University of Texas Medical Branch, Galveston, USA; 2 Department of Orthopedic Surgery, University of Texas Medical Branch, Galveston, USA

**Keywords:** closed reduction, finger phalanges, fracture dislocation, interphalangeal joints, volar avulsion fracture

## Abstract

A 77-year-old, right-hand-dominant woman presented after a low-energy fall with dorsal displacement involving the proximal and distal interphalangeal joints of the right small finger, along with a volar plate avulsion fracture at the base of the middle phalanx. Closed reduction was performed without anesthesia, followed by static aluminum splinting and occupational therapy. At her two-month follow-up, she reported minimal pain, improved function, and maintained joint alignment. This case highlights successful conservative management of a rare double interphalangeal dislocation with associated fracture in an elderly patient. It supports nonoperative treatment with early mobilization as a viable approach even in older adults with complex hand injuries.

## Introduction

Interphalangeal joint dislocations are common injuries in hand trauma, typically affecting a single joint. In contrast to the typical single-joint presentation, it is unusual for both interphalangeal joints of the same finger to be disrupted during a single injury [1-5]. They are most prominent in the fifth digit due to minimal tissue surrounding the joint, greater joint motion, limited stabilization from adjacent digits, and relatively weak capsuloligamentous support [2,5]. Radiographically, this injury presents as a *step ladder deformity* [3]. The proximal interphalangeal (PIP) and distal interphalangeal (DIP) joints are critical for grip, and simultaneous disruption of both joints can impair overall hand function.

Previously reported cases have predominantly occurred in young, male patients engaging in high-energy sports such as basketball or football and were successfully managed through closed reduction and brief immobilization [1,3,4,6-9]. While isolated reports in older adults exist, cases involving elderly women with associated osseous injuries, such as a volar plate avulsion fracture at the base of the middle phalanx, are exceedingly rare, and none have clearly documented this specific injury combination [10].

This report describes a unique case of a 77-year-old woman with concurrent PIP and DIP joint dislocations and a volar plate avulsion fracture at the base of the middle phalanx. Despite the complexity of this injury, it was treated successfully with closed reduction. The objective of this report is to contribute to the limited literature by highlighting a successful conservative approach in an elderly patient with a rare dislocation-fracture pattern, expanding the demographic scope, and reinforcing the viability of nonoperative care in older individuals. The patient gave verbal informed consent for details from her case to be published.

## Case presentation

**Figure 1:**
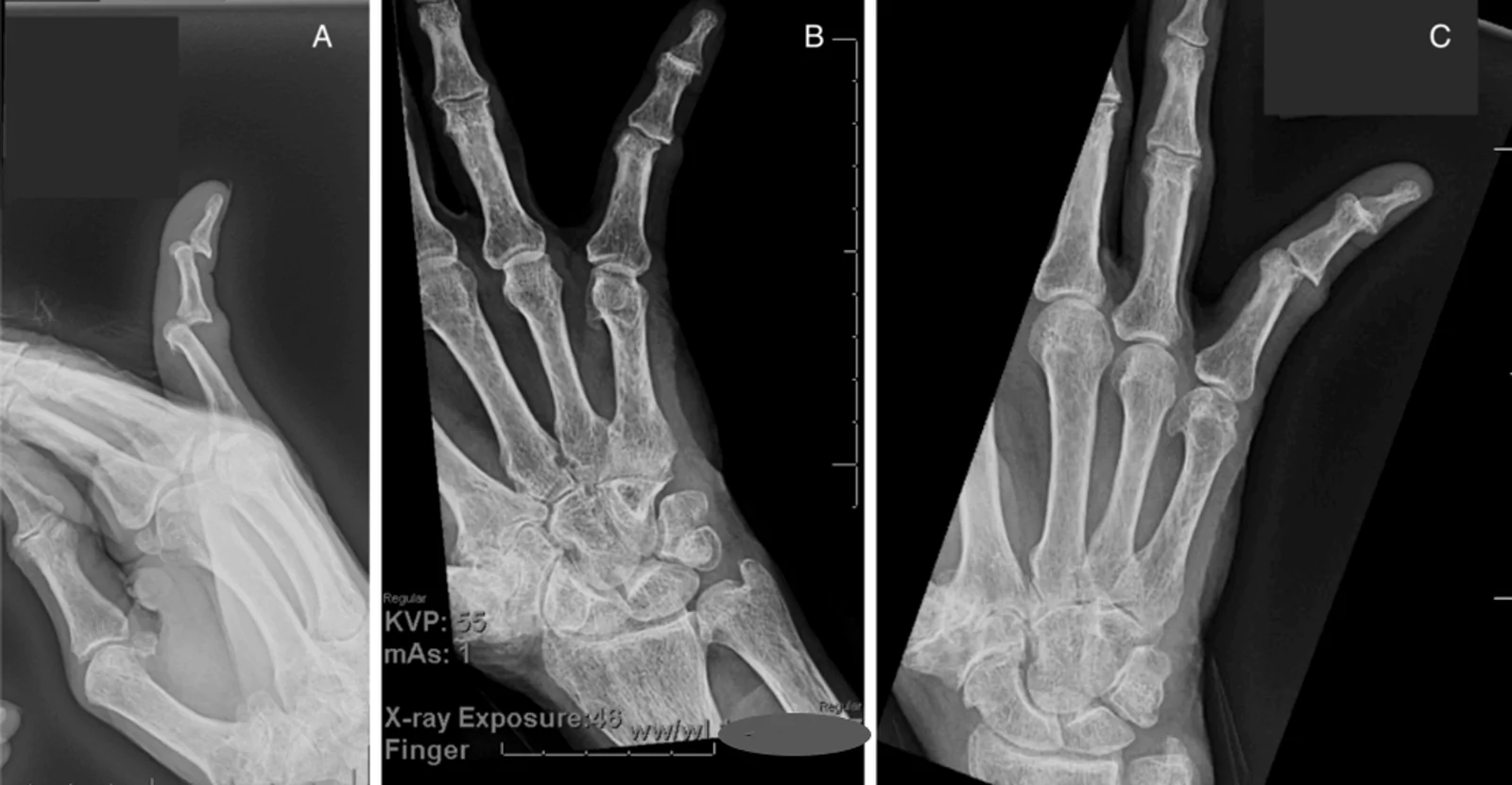
Lateral (A), anteroposterior (B), and oblique (C) radiographs demonstrating dorsal displacement at the PIP and DIP joints of the right fifth finger. PIP, proximal interphalangeal; DIP, distal interphalangeal

**Figure 2:**
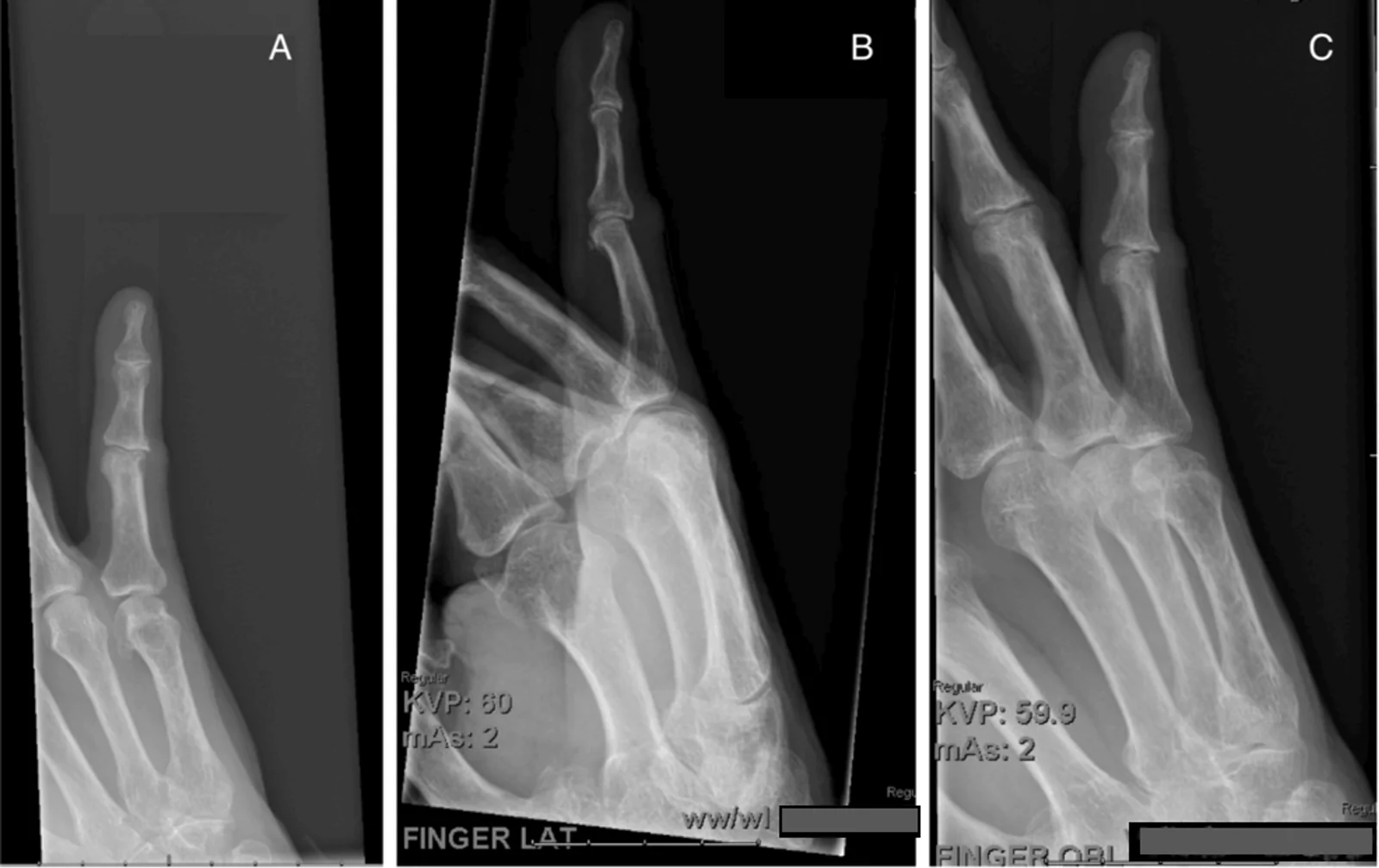
Post-reduction anteroposterior (A), lateral (B), and oblique (C) X-rays showing a 2-mm fracture fragment volar to the head of the right fifth proximal phalanx, with an apparent donor site at the base of the volar middle phalanx.

A 77-year-old, right-hand-dominant woman presented to the emergency room after falling and *jamming *her right fifth finger. She reported pain with attempted movement and deep palpation, but she denied numbness or paresthesia. On examination, the right fifth digit demonstrated visible deformity with dorsal displacement at both the PIP and DIP joints, associated swelling, and limited range of motion. Sensation was intact, and capillary refill was normal, indicating preserved neurovascular status. Radiographs demonstrated dorsal malalignment at the PIP and DIP joints of the right fifth digit with a suspected avulsion fracture at the metacarpal head (Figure 1). Closed reduction of both joints was successfully performed using direct traction without anesthesia. Post-reduction imaging confirmed successful alignment and revealed a 2-mm volar fracture fragment at the base of the middle phalanx (Figure 2). The digit was immobilized with a static aluminum finger splint, and the patient was advised to wear the splint continuously for one week. She was discharged with acetaminophen 650 mg every six hours as needed and instructed to follow up with her primary care provider.

She returned to the orthopedic clinic approximately four weeks post-injury. At that time, she had discontinued the splint and reported stiffness with minimal range of motion at the interphalangeal joints. Her score on the Quick Disabilities of the Arm, Shoulder, and Hand questionnaire (QuickDASH), a validated 11-item patient-reported outcome measure of upper extremity function and symptoms, was 50 [11]. Occupational therapy was initiated for range of motion, and she was instructed on buddy taping and the potential use of an extension block splint. Activity modification and non-steroidal anti-inflammatory drugs (NSAIDs) were recommended to manage pain, and she was advised to apply heat therapy before exercise.

**Figure 3:**
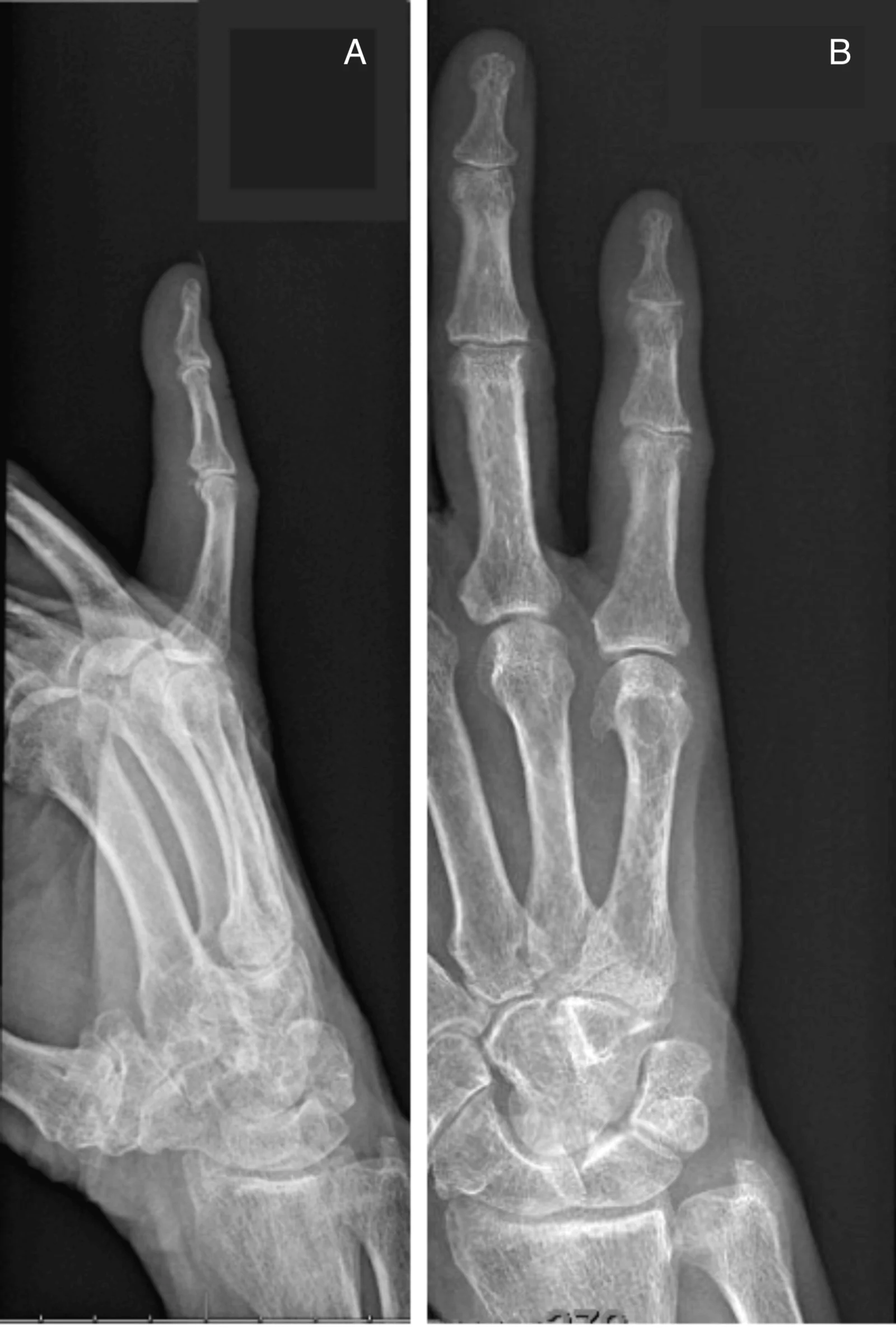
Lateral (A) and anteroposterior (B) X-ray at the two-month follow-up appointment.

At her follow-up two months post-injury, she reported decreased pain with daily activities, though she continued to have discomfort with more forceful hand use, such as opening jars or carrying groceries. Her QuickDASH score improved to 38.64. Repeat imaging showed maintained joint alignment and evidence of fracture healing (Figure 3). The joints remained stable without evidence of re-dislocation on subsequent evaluation. She was instructed to continue occupational therapy as needed, and follow-up was also scheduled on an as-needed basis. At her one-year follow-up, the patient was doing well but noted some mild arthritic pain in the DIP, PIP, and metacarpophalangeal joints of the right small finger. It did not limit her activities, but it did cause some pain with overuse. At 14 months post-injury, her QuickDASH score was 56.82, reflecting mild residual functional limitation despite overall satisfactory recovery.

## Discussion

Double dislocation of the interphalangeal joints within the finger is considered a rare injury. Previous literature suggests that these cases occur most commonly in athletes with sports-related trauma or in men around the age of 30 [5]. In contrast, our patient - a 77-year-old woman who sustained this injury from a low-energy fall - represents a markedly different demographic and mechanism, making this case particularly notable.

The mechanism of this injury occurs with hyperextension when an axial force is applied that ruptures the volar plate and displaces the distal phalanx dorsally. If the force is strong enough, it can then transmit to the PIP joint, causing a second dislocation [2,12]. This scenario was likely the cause of the patient's fall, which landed her on her dominant (right) hand.

It is important to treat these injuries rapidly to minimize the risk of circulatory impairment of the finger [10]. Conservative and surgical treatment options are available depending on the nature of the injury. Closed reduction is the treatment of choice in most cases, as it reduces the risk of complications and preserves the range of motion [5]. Early mobilization is important to prevent stiffness. However, for more complicated presentations, including soft-tissue damage and some cases of volar fracture, open surgery can provide greater stability in the long term [5].

Multiple case reports have demonstrated the success of conservative treatment. For instance, Kim et al. reported a case in which both the PIP and DIP joints were dislocated in the same finger and managed with closed reduction, with full recovery and no long-term sequelae [8]. Additionally, Takami et al. described three cases of double interphalangeal joint dislocations treated nonoperatively with excellent functional outcomes [4]. These cases reinforce the efficacy of closed reduction and early mobilization, particularly in the absence of soft-tissue interposition or large fracture fragments. However, not all cases are straightforward. Bamal and Bindra described neglected PIP dislocations where soft-tissue contracture limited the success of closed reduction, necessitating open repair to restore stability and range of motion [13]. Kolovich et al. and Haase et al. reviewed the treatment of PIP fracture-dislocations, emphasizing that when fracture fragments compromise joint stability, surgical intervention may be required [14,15]. These findings underscore that patient age, chronicity, and the presence of bony injury all influence treatment decisions.

To our knowledge, this study is one of the few reported cases of simultaneous PIP and DIP dislocations with an associated volar plate avulsion fracture at the base of P2 in an elderly woman managed conservatively with excellent outcome. This case expands the known demographic and injury profiles and reinforces that nonoperative management remains a viable and effective strategy, even in older individuals with complex dislocation-fracture patterns. It also highlights the importance of timely recognition and individualized treatment planning regardless of age or injury mechanism.

In our case, post-reduction imaging revealed a 2-mm volar fracture fragment consistent with a volar plate avulsion fracture at the base of the middle phalanx. Despite the added complexity of this fracture and the patient’s advanced age, she was successfully managed with closed reduction, splinting, and early mobilization, ultimately achieving near-full functional recovery. As a single-case report, this study is limited in its ability to establish generalizable conclusions, and findings should be interpreted within the context of existing literature.

## Conclusions

This case illustrates an abnormal, complex, dorsal displacement at both interphalangeal joints of the right small finger with an associated volar fracture in a 77-year-old woman. Unlike previously reported cases, typically involving young, athletic males with high-energy trauma, this injury resulted from a low-energy fall and was successfully managed with closed reduction, splinting, and early mobilization. At the two-month follow-up, the patient demonstrated satisfactory healing and improved functional recovery, despite her advanced age and the presence of a fracture. This case adds to the known demographic and injury profiles and reinforces that nonoperative management remains a viable and effective strategy, even in older individuals with complex dislocation-fracture patterns.
